# Simufilam Reverses Aberrant Receptor Interactions of Filamin A in Alzheimer’s Disease

**DOI:** 10.3390/ijms241813927

**Published:** 2023-09-11

**Authors:** Hoau-Yan Wang, Erika Cecon, Julie Dam, Zhe Pei, Ralf Jockers, Lindsay H. Burns

**Affiliations:** 1 Department of Molecular, Cellular and Biomedical Sciences, City University of New York School of Medicine, New York, NY 10031, USA; hwang@med.cuny.edu (H.-Y.W.); zpei@ccny.cuny.edu (Z.P.); 2 Department of Biology and Neuroscience, Graduate School, City University of New York, New York, NY 10016, USA; 3 Institut Cochin, INSERM, CNRS, Université Paris Cité, 75014 Paris, France; erika.cecon@inserm.fr (E.C.); julie.dam@inserm.fr (J.D.); ralf.jockers@inserm.fr (R.J.); 4 Cassava Sciences, Inc., Austin, TX 78731, USA

**Keywords:** α7 nicotinic acetylcholine receptor, TLR4, TLR2, CXCR4, CD4, CCR5, TR-FRET

## Abstract

Simufilam is a novel oral drug candidate in Phase 3 clinical trials for Alzheimer’s disease (AD) dementia. This small molecule binds an altered form of filamin A (FLNA) that occurs in AD. This drug action disrupts FLNA’s aberrant linkage to the α7 nicotinic acetylcholine receptor (α7nAChR), thereby blocking soluble amyloid beta_1–42_ (Aβ_42_)’s signaling via α7nAChR that hyperphosphorylates tau. Here, we aimed to clarify simufilam’s mechanism. We now show that simufilam reduced Aβ_42_ binding to α7nAChR with a 10-picomolar IC_50_ using time-resolved fluorescence resonance energy transfer (TR-FRET), a robust technology to detect highly sensitive molecular interactions. We also show that FLNA links to multiple inflammatory receptors in addition to Toll-like receptor 4 (TLR4) in postmortem human AD brains and in AD transgenic mice: TLR2, C-X-C chemokine receptor type 4 (CXCR4), C-C chemokine receptor type 5 (CCR5), and T-cell co-receptor cluster of differentiation 4 (CD4). These aberrant FLNA linkages, which can be induced in a healthy control brain by Aβ_42_ incubation, were disrupted by simufilam. Simufilam reduced inflammatory cytokine release from Aβ_42_-stimulated human astrocytes. In the AD transgenic mice, CCR5–G protein coupling was elevated, indicating persistent activation. Oral simufilam reduced both the FLNA–CCR5 linkage and the CCR5–G protein coupling in these mice, while restoring CCR5′s responsivity to C-C chemokine ligand 3 (CCL3). By disrupting aberrant FLNA–receptor interactions critical to AD pathogenic pathways, simufilam may promote brain health.

## 1. Introduction

Alzheimer’s disease (AD) is the most common neurodegenerative disease and the most common form of dementia, with over 55 million cases worldwide and expected to double every 20 years, underscoring the need for effective disease-modifying treatments [1]. In the U.S., there are 6.7 million people living with AD with an additional 11 million family and friends caring for them [2], totaling 5.3% of the U.S. population. 

The FDA has recently approved two anti-amyloid antibody therapies for patients with early AD. These infusion drugs are celebrated as nominal successes, tempered by their modest impact on disease progression, a black box cautionary warning regarding cerebral hemorrhages, the possible need for APOE genotyping and PET scans, the requirement for frequent MRIs to monitor drug-induced brain swelling and brain bleeding, and the inconveniences and exceptional expense of drug infusion therapy, which also limit access to rural or underserved populations [3]. More recently noted is the possible shrinkage of brain volume over time, which is not fully understood [4]. Adding complexity, drug effectiveness may vary by gender and APOE genotype [5] and degree of tau deposition [6]. Finally, the regulatory use of these infusion drugs is restricted to patients with early AD, i.e., mild cognitive impairment and mild AD.

Alternatives to anti-amyloid therapies are sorely needed. Those being investigated clinically include agents targeting tau, neuroinflammation, synaptic plasticity, metabolism or proteostasis [7]. Simufilam is a novel oral drug candidate with preclinical data showing reduced tau hyperphosphorylation and neurofibrillary tangles, reduced neuroinflammation, improved synaptic plasticity and improved insulin receptor signaling. We posit that all these beneficial effects are downstream to restoring the normal conformation of simufilam’s target protein, altered FLNA [8,9,10]. 

FLNA is a large intracellular scaffolding protein known to interact with over 90 different proteins [11]. It contains 24 immunoglobulin-like repeats, two hinge regions and two rod domains [12,13]. The 24th repeat dimerizes in the membrane to form a V shape inside the cell. Best known for cross-linking actin via the N-terminal domain to provide structure and motility, FLNA also serves as a scaffold for channels, receptors, signaling molecules and even transcription factors, illustrating a role beyond structure [11,14,15]. FLNA is highly expressed in the brain, and its protein interactions are regulated by mechanical forces, phosphorylation, cleavage and other factors [11,13,16,17]. 

An altered conformation of FLNA would likely alter certain protein interactions or induce aberrant ones. A region of FLNA unfolds under forces as low as 10 pN [17], and stress-induced conformational changes have been hypothesized to play a direct role in signaling, either by disrupting existing interactions or inducing new ones [18]. In an altered conformation implied by a shift in isoelectric focusing point [8,10,19] and a change in solubility [16], FLNA appears to be a critical and deviant receptor-associated protein underlying multiple facets of AD pathology [9,10]. Specifically, deviant FLNA linkages are critical to Aβ_42_-induced tau hyperphosphorylation, leading to neurodegeneration, and to Aβ_42_-induced activation of TLR4, leading to neuroinflammation [9,10]. The disruption of these aberrant receptor interactions by simufilam is coincident with a reversal of the shift in isoelectric focusing, implying a reversion to FLNA’s native shape [8,10].

Simufilam’s primary mechanism is to disrupt the toxic signaling of soluble Aβ_42_ via the α7nAChR that hyperphosphorylates tau [9,10,20]. The ultra-high-affinity binding of Aβ_42_ for α7nAChR was first published in 2000 [21,22], and this Aβ_42_–α7nAChR interaction was later shown by Wang and other researchers to activate kinases that hyperphosphorylate tau [23,24,25,26]. 

Hyperphosphorylated tau can no longer stabilize microtubules, impairing intraneuronal transport of proteins, which causes the accumulation of hyperphosphorylated tau aggregates, eventual neurodegeneration and tau-containing tangles [27,28,29]. As increasing soluble Aβ_42_ piles onto this receptor, the Aβ_42_–α7nAChR complex is internalized into the cell by endocytosis, leading to intraneuronal amyloid aggregates and eventual amyloid deposits or dense-core plaques after cell death [30,31]. Hence, this pathogenic signaling pathway of soluble Aβ_42_ mechanistically links the hallmark plaques and tangles [30,32,33]. 

Simufilam dismantles this prominent AD pathogenic pathway by disrupting the linkage of FLNA with α7nAChR, an interaction critical both to the toxic signaling and to the ultra-high-affinity binding of Aβ_42_ [9]. By disrupting this pathway in the AD brain, simufilam slows or reduces neurodegeneration.

This aberrant FLNA–α7nAChR linkage can be induced in normal tissue by incubation with Aβ_42_, along with the shift in isoelectric focusing point that implies an altered conformation of FLNA [10], and both are reversible by simufilam [8,10]. By disrupting the FLNA–α7nAChR linkage and restoring the native FLNA conformation, simufilam reduced the femtomolar binding affinity of Aβ_42_ for α7nAChR 1000-fold in postmortem brain synaptic membranes and 10,000-fold in SK-N-MC cells [9]. In the current work, we used a cell-based TR-FRET assay [34] to confirm that simufilam reduces Aβ_42_ binding to α7nAChR. 

The second pathogenic signaling pathway of soluble amyloid that is disrupted by simufilam is Aβ_42_’s persistent activation of TLR4 by Aβ_42_ binding to the TLR4 co-receptor CD14 [35]. TLR4’s activation by Aβ_42_ requires the aberrant linkage of FLNA with TLR4 [8,9,10]. In a similar mechanism, simufilam disrupts the FLNA–TLR4 linkage to suppress the persistent activation of this receptor and resulting inflammatory cytokine release to suppress neuroinflammation [8,9,10]. 

Because neuroinflammation is a prominent AD pathology [36], we explored whether Aβ_42_ may induce FLNA linkages with other inflammatory receptors found on microglia that are involved in a persistent inflammatory response. TLR2 was selected as it is also activated by Aβ_42_ [37], but, unlike TLR4, does not use the CD14 co-receptor for activation and produces different cytokines and chemokines [38]. The chemokine receptors CXCR4 and CCR5 and T-cell receptor CD4 were selected because they act synergistically or step-wise in inflammation. CCR5 is a prominent chemokine receptor upregulated on microglia in AD [39]. CXCR4 and CD4 are also expressed on microglia and often cluster with CCR5 [40]. 

We assessed whether ex vivo simufilam incubation of postmortem human AD brains or oral administration of simufilam to triple transgenic AD mice could disrupt these additional aberrant FLNA–receptor linkages. Next, to determine whether the FLNA linkages with these receptors may indicate receptor activation and whether simufilam could suppress their activation by disrupting the FLNA–receptor linkages, we tested simufilam’s effects on inflammatory cytokine release in human astrocytes stimulated in vitro with Aβ_42_, lipopolysaccharide (LPS; an activator of TLR4), or TLR2 ligands: lipoteichoic acid from Staphylococcus aureus (LTA-SA) and peptidoglycan from Staphylococcus aureus (PGN-SA). Finally, in the brains of the AD transgenic mice, we examined whether the FLNA linkage with CCR5, a G-protein-coupled receptor, was coincident with elevated G protein coupling by CCR5, which would indicate persistent CCR5 activation and potentially an insensitivity to CCR5’s natural ligand CCL3. The AD transgenic mice administered oral simufilam in drinking water allowed for the assessment of simufilam’s effects on persistent CCR5 activation and dysfunction.

## 2. Results

### 2.1. Simufilam Reduced Aβ_42_ Binding to α7nAChR

**Figure 1:**
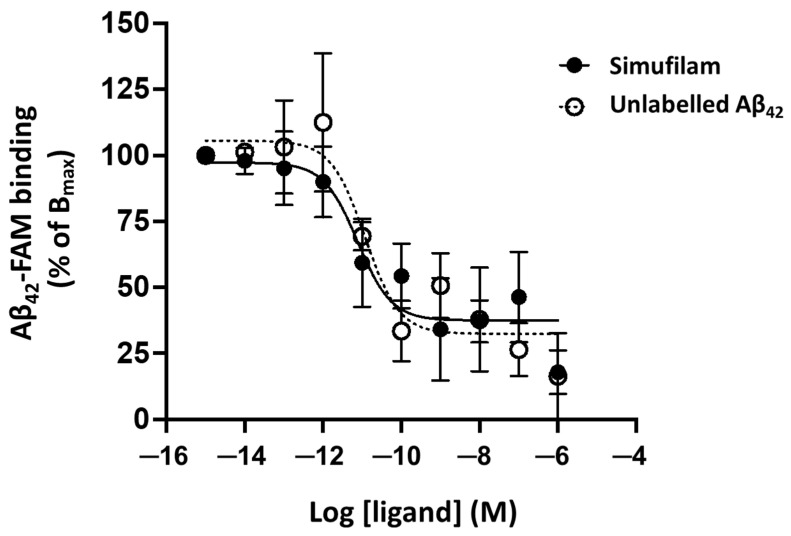
Simufilam reduced Aβ_42_ binding to α7nAChR in a TR-FRET assay. Aβ_42_-FAM binding to SNAP-α7nAChR in HEK293T cells was measured in the presence of increasing concentrations of simufilam or unlabeled Aβ_42_. Data are means of pooled data from 4 separate experiments ± SEM.

The effect of simufilam on Aβ_42_ binding to α7nAChR was determined by a TR-FRET assay, which relies on the excitation of Aβ_42_-FAM (donor fluorophore) to produce an energy transfer to SNAP-α7nAChR (acceptor fluorophore) if they are in close proximity (<10 nm; Förster radius). Simufilam reduced Aβ_42_ binding to α7nAChR in a concentration-dependent manner, with a mean IC_50_ of four separate experiments in the pM range (pIC_50_ = 10.9 ± 0.5 or 12.6 pM when converted to molarity) (Figure 1). By comparison, the mean IC_50_ for unlabeled Aβ_42_ was also in the low pM range (pIC_50_ = 11.9 ± 0.5 or 1.3 pM). Because simufilam does not directly interact with either Aβ_42_ or α7nAChR, its reduction in Aβ_42_ binding to α7nAChR in this assay is hypothesized to occur by dissociating FLNA from the Aβ_42_–α7nAChR complex, thereby releasing Aβ_42_ in a concentration-dependent manner. This result corroborates our earlier demonstration that simufilam reduces Aβ_42_ affinity (increasing off rate) for α7nAChRs in SK-N-MC cells and postmortem human brains [9].

### 2.2. Simufilam Reduced FLNA–TLR2 Linkage and Cytokine Release Stimulated by Aβ_42_ and TLR2 Agonists

**Figure 2:**
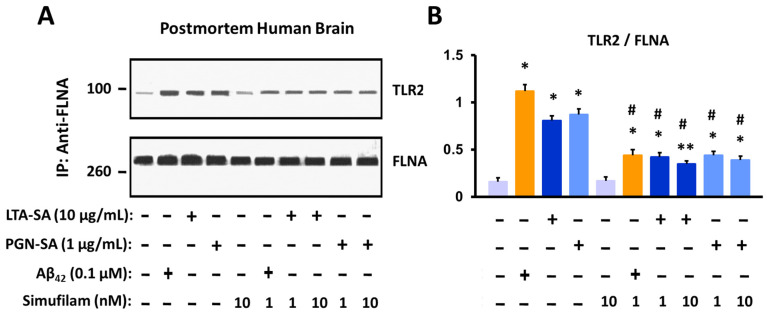
Incubation of postmortem human frontal cortex with TLR2 ligands or Aβ_42_ increases FLNA linkage with TLR2. This FLNA—TLR2 linkage is inhibited by simufilam at 1 or 10 nM. Representative blots (**A**) and densitometric quantitation of blots (**B**). Data are means ± SEM. N = 3. * *p* < 0.001, ** *p* < 0.01 vs. medium alone; # *p* < 0.01 vs. respective stimulant without simufilam.

Because FLNA also links to TLR4, allowing Aβ_42_’s chronic activation of this receptor via its co-receptor CD14, we next examined whether FLNA might also interact with TLR2, which is stimulated by Aβ_42_ directly [37]. Incubation of a control postmortem human frontal cortex with Aβ_42_ or the TLR2 ligands (LTA-SA or PGN-SA) dramatically elevated the levels of FLNA linkage to TLR2 (*p* < 0.001; Figure 2). Simufilam incubation at 1 or 10 nM reduced these FLNA–TLR2 linkages induced by Aβ_42_ or the TLR2 agonists (*p* < 0.01). The similar effects of 1 and 10 nM simufilam suggest that 1 nM is a saturating concentration and is in accordance with the picomolar IC_50_ demonstrated for reducing Aβ_42_ binding to α7nAChR.

The co-immunoprecipitation experiments to determine protein–protein interactions were conducted with synaptosomes, i.e., sealed presynaptic terminals that can be prepared in high yield (~80%) from brain tissue, which have been used since the 1960s [41,42,43,44] and specifically to examine synaptic terminals in AD brain tissue [45]. 

**Figure 3:**
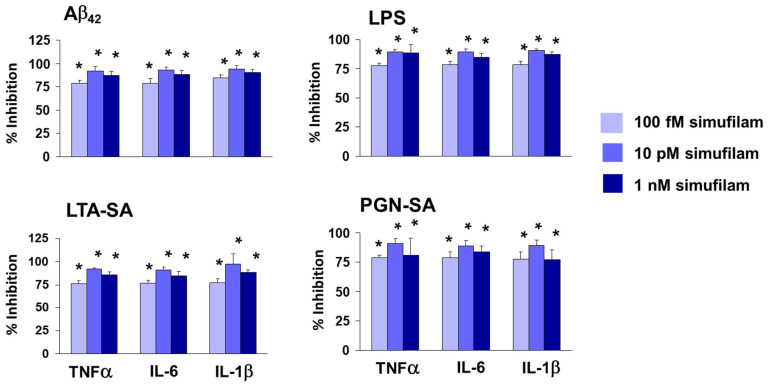
Simufilam inhibits release of inflammatory cytokines by human astrocytes stimulated with Aβ_42_, LPS or TLR2 ligands LTA-SA and PGN-SA. Data are means ± SEM. N = 3. * *p* < 0.001 simufilam vs. respective stimulant alone.

To assess whether the FLNA linkage represents activation of TLR2 by these ligands and whether its disruption might reduce such activation, we measured cytokine release from human astrocytes stimulated for 24 h with Aβ_42_, the TLR2 agonists or LPS (a TLR4 activator) and measured the effect of simufilam, added 2 h prior to the stimulants, on the cytokine release. Simufilam at 100 fM, 10 pM or 1 nM reduced the release of inflammatory cytokines tumor necrosis factor α (TNFα), interleukin (IL)-6 and IL-1β by approximately 75% or more (*p* < 0.001; Figure 3). It is possible that the lack of concentration response in this experiment is related to the 2 h pre-treatment with simufilam prior to the 16 h incubation with the stimulants, favoring simufilam’s prevention of cytokine release.

### 2.3. Simufilam Reduced FLNA–CXCR4/CD4/CCR5 Linkages

**Figure 4:**
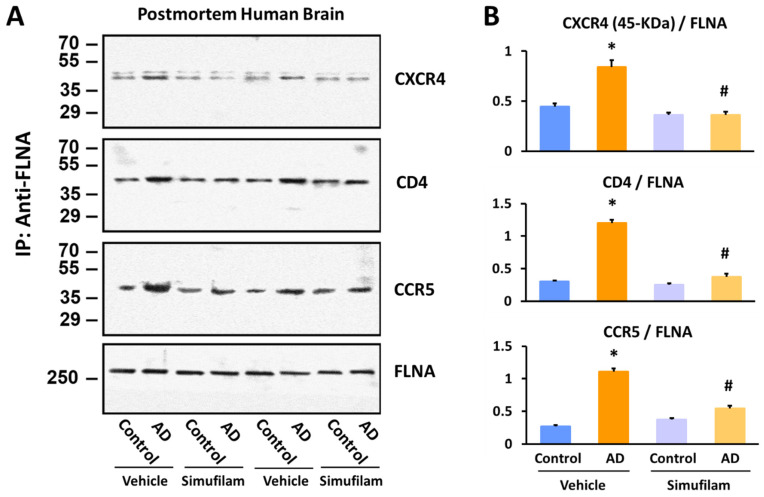
Simufilam incubation (1 nM for 1 h) reduced FLNA linkages with CXCR4, CD4 and CCR5 in AD postmortem brain to levels not different from healthy control brain. Representative blots (**A**) and densitometric quantitation of blots (**B**). Data are means ± SEM. N = 11. * *p* < 0.001 AD vs. control brain tissue incubated with vehicle; # *p* < 0.01 simufilam vs. vehicle incubation of AD brain tissue.

We next broadened our investigation to additional inflammatory receptors: the chemokine receptors CXCR4 and CCR5 and the T cell co-receptor CD4. For FLNA linkages to all three receptors in synaptosomes from AD versus age-, gender- and postmortem-interval-matched healthy control brain tissue, two-way ANOVAs showed highly significant main effects of diagnosis (CXCR4: F = 22.30, *p* < 0.0001; CD4: F = 188.52, *p* < 0.0001; CCR5: F = 179.43, *p* < 0.0001) and treatment (CXCR4: F = 44.39, *p* < 0.0001; CD4: F = 140.48, *p* < 0.0001; CCR5: F = 35.78, *p* < 0.0001) and a diagnosis–treatment interaction (CXCR4: F = 22.29, *p* < 0.0001; CD4: F = 109.68, *p* < 0.0001; CCR5: F = 79.78, *p* < 0.0001). In postmortem AD brain tissue, FLNA linkages to these three receptors were elevated compared to levels in non-demented control brain tissue (*p* < 0.001; Figure 4). Simufilam incubation of brain tissue (1 nM for 1 h) significantly reduced these elevated linkages in AD brain synaptosomes (*p* < 0.01), while having no effect on the lower levels in control brain synaptosomes. 

**Figure 5:**
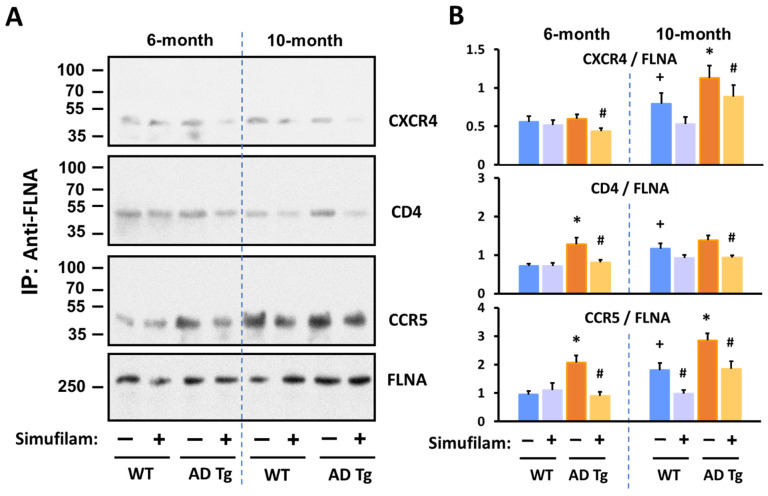
Simufilam reduced FLNA linkages with CXCR4, CD4 and CCR5 in AD triple transgenic mouse brains. Simufilam (22 mg/kg/d) was administered via drinking water for 2 months, starting at 4 months or at 8 months. Simufilam also reduced the slightly lower levels of these FLNA linkages found in 10-month wildtype mice. Representative blots (**A**) and densitometric quantitation of blots (**B**). Data are means ± SEM. N = 5. * *p* < 0.001 AD Tg vs. wildtype; # *p* < 0.001 simufilam vs. water alone in respective age transgenic mice; + *p* < 0.001 vs. 6-month wildtypes.

We also examined FLNA linkages with CXCR4, CD4 and CCR5 in synaptosomes from AD triple transgenic mice versus wildtype mice at 6 or 10 months of age after 2 months of oral simufilam via drinking water (Figure 5). We selected 4 months and 8 months to initiate treatment, as these ages correspond to pre-plaque and post-plaque pathology in this transgenic line. The dose of 22 mg/kg/d was based on a prior experiment using 10 mg/kg b.i.d. by i.p. infusion in an acute AD mouse model [9] and the drug’s high oral bioavailability. 

FLNA–CXCR4 was significantly elevated in 10-month (but not 6-month) transgenic mice vs. wildtypes (*p* < 0.001). FLNA–CD4 was significantly elevated in 6-month transgenics versus wildtypes (*p* < 0.001) but was not significantly different in 10-month transgenic vs. the 10-month wildtypes due to the higher levels of this linkage in the older versus younger wildtypes. FLNA–CCR5 was elevated in transgenics of both ages relative to respective aged wildtypes (*p* < 0.001). All three FLNA linkages were also significantly elevated in the 10-month versus 6-month wildtype mice (*p* < 0.001). Importantly, 2-month oral simufilam treatment significantly reduced FLNA linkages with all three receptors in the transgenics of both ages as well as the FLNA–CCR5 linkage in the 10-month wildtypes (*p* < 0.001).

### 2.4. Simufilam Reduced Chronic CCR5 Activation in AD Transgenic Mice

To confirm that the FLNA linkage with CCR5 results in CCR5 activation, we measured the level of CCR5–G protein coupling in the transgenic mice given drinking water with or without simufilam for 2 months. Basal (unstimulated) G protein coupling by CCR5 was assessed in synaptic membranes of these mice, and this CCR5–G protein coupling was also measured following stimulation of synaptic membranes with the CCR5 ligand CCL3. 

**Figure 6:**
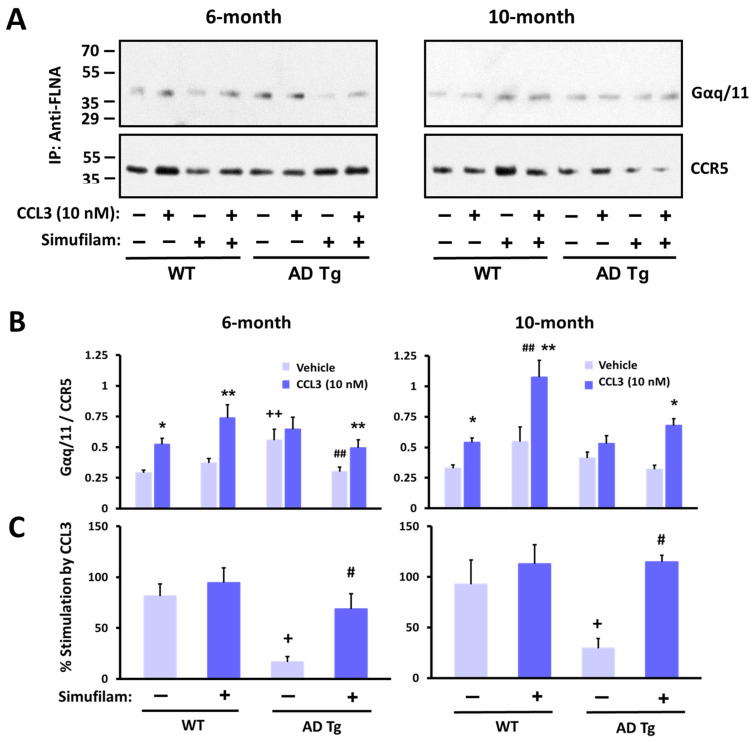
CCR5 coupling to G protein Gaq/11 was elevated in AD transgenic mice versus wildtypes, with little stimulation by the CCR5 ligand CCL3. Oral simufilam (22 mg/kg/d) for 2 months, starting at 4 months or at 8 months, reduced the elevated CCR5–G protein coupling and improved CCL3-induced G protein coupling of CCR5. Representative blots (**A**) densitometric quantitation (**B**) and percent stimulation by CCL3 (**C**). Data are means ± SEM. N = 5 (except N = 4 for 10-month-old wildtypes administered vehicle). * *p* < 0.01, ** *p* < 0.05 CCL3 vs. vehicle in the same group; # *p* < 0.01, ## *p* < 0.05 simufilam vs. vehicle in respective age transgenic/wildtype; + *p* < 0.01, ++ *p* < 0.05 transgenic vs. respective age wildtype.

Levels of unstimulated CCR5-coupled Gq/11 protein were elevated in 6-month transgenics compared to wildtypes (Figure 6B; *p* < 0.05), suggesting chronic activation. However, the basal coupling in 10-month transgenics was not significantly higher than basal CCR–G protein coupling in the older wildtypes. Stimulation with CCL3 did not further increase G protein coupling in transgenics of either age. In contrast, the wildtype mice of both ages showed a significant increase in CCR5–G protein coupling after stimulation by CCL3 (*p* < 0.01). Percent stimulation by CCL3 in transgenics was significantly lower than in wildtypes (Figure 6C; *p* < 0.01 for both ages). 

Simufilam oral treatment for 2 months restored the response to CCL3 in transgenics, primarily by reducing basal levels to that of wildtype controls (Figure 6B; *p* < 0.05 in 6-month transgenics). Although simufilam did not significantly reduce basal CCR5–G protein coupling in 10-month transgenics or significantly enhance the response to CCL3 in these older transgenics, the CCL3 response was significant in simufilam-treated but not vehicle-treated transgenics of both ages (CCL3 vs. vehicle for 6-month transgenics treated with simufilam: *p* < 0.05; CCL3 vs. vehicle for 10-month transgenics treated with simufilam: *p* < 0.01). Interestingly, the absolute CCL3-induced Gq/11 coupling to CCR5 was higher in 10-month simufilam- vs. vehicle-treated wildtypes (*p* < 0.05). Simufilam also improved the percent stimulation by CCL3 in transgenics of both ages (Figure 6C; *p* < 0.01). 

## 3. Discussion

This work further elucidates the mechanism of action of oral AD drug candidate simufilam, i.e., reducing both neurodegeneration and neuroinflammation [8,9,10,20]. We previously showed that simufilam oral treatment or ex vivo incubation of brain tissue reduced levels of Aβ_42_–α7nAChR and FLNA–α7nAChR complexes [8,9,10]. We now show that simufilam reduced the binding of Aβ_42_ to α7nAChR in a concentration-dependent manner using TR-FRET, a robust technology for the detection of molecular interactions that are highly sensitive to conformational modifications [46]. 

The 10 pM IC_50_ of simufilam in inhibiting the binding of Aβ_42_ to α7nAChR in this assay was only 10-fold lower than the 1 pM IC_50_ of unlabeled Aβ_42_ (direct competition) and similar to the pIC_50_s of several agonists, partial agonists or competitive antagonists of α7nAChR (range: 8.4 to 12.7 pIC_50_) [34]. Notably, full and partial agonists of α7nAChR were only able to reduce Aβ_42_ binding by 66–83% of the full inhibition by unlabeled Aβ_42_, and only methylylcaconitine, a competitive antagonist, was able to inhibit the Aβ_42_–α7nAChR interaction to the full extent of unlabeled Aβ_42_. Inhibition in this TR-FRET assay was not seen with a non-competitive antagonist or a type 1 positive allosteric modulator of α7nAChR [34]. Simufilam’s low picomolar IC_50_ and magnitude of inhibition very close to that of unlabeled Aβ_42_ are unprecedented for its mechanism of binding a receptor-associated protein. 

These TR-FRET data corroborate simufilam’s reduction in Aβ_42_’s binding affinity of Aβ_42_ for α7nAChR shown by FITC-labeled Aβ_42_ in postmortem human brain and in fresh SK-N-MC cells [9]. The picomolar IC_50_ also agrees with picomolar IC_50_s for simufilam’s inhibition of the Aβ_42_–α7nAChR interaction, tau hyperphosphorylation, and FLNA–α7nAChR/TLR4 interactions calculated for a range of concentrations in postmortem brain [10] and also shown in AD mouse models or AD patient lymphocytes [8,9,10]. Further support is that two other independent laboratories showed biological activity of simufilam in FLNA-related disorders [47,48]. Together, all these data support simufilam’s primary mechanism of reducing soluble Aβ_42_’s signaling that hyperphosphorylates tau. Disrupting Aβ_42_’s pathogenic signaling through α7nAChR would also promote healthy α7nAChR neurotransmission. 

Illustrating an additional AD-relevant mechanism of action, simufilam also disrupts an aberrant linkage of FLNA with TLR4, which again is induced by soluble Aβ_42_ binding, in this case to TLR4’s co-receptor CD14 [8,9,10]. Extending the anti-neuroinflammatory mechanism of action of simufilam, we now show that simufilam reduced the Aβ_42_-induced FLNA interactions with additional inflammatory receptors: TLR2, the chemokine receptors CXCR4 and CCR5, and T-cell co-receptor CD4. Postmortem human frontal cortexes from non-demented controls showed FLNA interactions with TLR2 induced by Aβ_42_ or TLR2 agonists; simufilam reduced these linkages. Simufilam’s 75% or greater reductions in inflammatory cytokine release from primary human astrocytes stimulated with Aβ_42_ or TLR2/TLR4 agonists suggest that the FLNA–receptor linkages, which are reduced by simufilam, are critical to agonist activation of these receptors. 

Both postmortem human AD brain tissue and triple transgenic AD mouse brains showed elevated interactions of FLNA with CXCR4, CCR5 and CD4. Ex vivo simufilam incubation of the postmortem tissue or 2-month oral administration to the mice significantly reduced these linkages, suggesting that simufilam reduced inflammatory signaling. We previously showed that the brains of these same AD transgenic mice treated with 2-month oral simufilam showed reduced FLNA linkages with α7nAChR and TLR4, reduced tau hyperphosphorylation, reduced inflammatory cytokine levels, reduced amyloid deposits and neurofibrillary lesions, improved function of NMDA and insulin receptors, and improved activity-dependent Arc expression (an indicator of synaptic plasticity) [10]. All these drug effects were coincident with the isoelectric focusing point of FLNA shifting back to that of FLNA in wildtype control brains [10].

Finally, the elevated G protein coupling of CCR5 in the triple transgenic AD mice, along with CCR5’s insensitivity to further activation by its ligand CCL3 in these transgenics, provides additional evidence that elevated FLNA linkages to inflammatory receptors in AD imply their chronic activation and resulting neuroinflammation. Simufilam’s suppression of the elevated basal CCR5–G protein coupling and improvement to CCR5’s responsivity to its ligand CCL3 again support the hypothesis that simufilam reduces chronic activation of multiple inflammatory receptors in AD. Of note, Aβ_42_ also interacts with the A2A adrenergic receptor [49] and the leptin receptor [50] to modify basal or ligand-induced signaling pathways. 

Reducing activation of multiple inflammatory receptors would benefit AD. Indeed, 17% of AD therapeutic candidates currently in clinical trials target neuroinflammation [7]. Neuroinflammation in AD is not merely a reaction to plaques and tangles but contributes to disease progression and severity [51]. Although early microglial recruitment promotes clearance of soluble Aβ, as the disease progresses, elevated inflammatory cytokines can lead to insufficient phagocytic clearance of soluble Aβ, resulting in greater toxic signaling via α7nAChR and TLR4/2, intraneuronal Aβ accumulation, tau hyperphosphorylation and further inflammation, leading to extensive neurodegeneration [52,53]. 

The inflammatory cytokines TNFα, IL-1β and IL-17 can loosen tight junctions and compromise the blood–brain barrier [54], another pathological feature of AD, which enables an influx of immune cells to exacerbate neuroinflammation [55,56]. Because healthy microglia regulate synaptic pruning, synaptic plasticity and learning and memory, abnormal microglial activation and the resulting neuroinflammation have been causally implicated in the cognitive deficits of normal aging, AD and other diseases [57]. 

By suppressing neuroinflammation, simufilam may also reduce insulin resistance associated with AD: neuroinflammation in both AD and obesity or type 2 diabetes induces insulin resistance and insulin receptor dysfunction [58,59]. TNFα has been shown to induce insulin resistance [60,61]. Neuroinflammation is a critical link between AD, depression, and obesity, with each increasing risk of the others [62]. Indeed, simufilam has been shown to improve brain insulin receptor signaling [9,10]. Illustrating reduced insulin resistance, oral simufilam improved the response to insulin of mammalian target of rapamycin (mTOR) and suppressed mTOR’s basal overactivation in lymphocytes of AD subjects [63]. With insulin receptors critical for cell survival and cell health, reduced brain insulin resistance, if translating from the lymphocytes, would lessen this contribution to neurodegeneration.

In addition to the induced aberrant receptor interactions with α7nAChR and multiple inflammatory receptors, the altered conformation of FLNA in AD may impact FLNA’s normal protein interactions. We previously showed that FLNA normally interacts with the intracellular phosphatase PTEN and that this healthy FLNA interaction is reduced in AD [63]. There may be other aberrant protein interactions that are reduced and other normal protein interactions that are preserved by restoring FLNA’s native shape in AD brains. 

## 4. Materials and Methods 

### 4.1. Materials and Chemicals

Aβ_1–42_ human, LTA-SA and PGN-SA were obtained from Invitrogen. For TR-FRET assays, Aβ_1–42_ human and Aβ_42_-FAM were purchased from Anaspec (Fremont, CA, USA). Recombinant human CCL3/MIP-1 alpha protein was purchased from R&D Systems (Minneapolis, MN, USA). Anti-TLR2 (SC-166900), -CCR5 (SC-17833), -CD4 (SC-19641), and -CXCR4 (SC-53534), -FLNA (SC-7565 [IP], SC-17749 [IP], SC-271440), Gαq/11 (SC-515689), anti-tumor necrosis factor α (TNFα) (SC-8301), anti-Interleukin-6 (IL-6) (SC-7920), anti-Interleukin-1β (IL-1β) (SC-7884) were purchased from Santa Cruz Biotechnology (Santa Cruz, CA, USA). Reacti-Bind NeutrAvidin high-binding capacity coated 96-well plates, covalently conjugated protein A/G-agarose beads, antigen elution buffer and Chemiluminescent reagents were purchased from Pierce-Thermo Scientific (Rockford, IL, USA). Biotinylated anti-IL1β (13-7016-85), anti-TNFα (13-7349-85) and anti-IL-6 (13-7068-85) were purchased from eBioscience (San Diego, CA, USA). LPS, phosphatase inhibitors (Roche), complete mini ethylenediaminetetraacetic acid (EDTA)-free protease inhibitor tablet (Roche), and alkaline phosphatase were purchased from Sigma (St. Louis, MO, USA). Aβ-derived peptides were dissolved in 50 mM Tris, pH 9.0 containing 10% dimethyl sulfoxide (DMSO) and stored at −80 °C. All test agents were freshly made according to manufacturers’ recommendations. If DMSO was used as the solvent, the highest DMSO concentration in the incubation was 1%.

### 4.2. TR-FRET Binding Assay

Aβ_42_ binding to α7nAChR was monitored by a TR-FRET assay, as previously described [34]. Briefly, HEK293T cells were transfected to express SNAP-α7nAChR and the chaperone protein NACHO [64]. Forty-eight hours post-transfection, surface SNAP-α7nAChR was labeled with the long-lived fluorophore Terbium cryptate (Tb; Lumi4-Tb, Cisbio Bioassays, Codolet, France) by incubating cells with the Tb-conjugated SNAP substrate in Tag-lite labeling medium (100 nM, 1 h, 4 °C). After 3 washes in PBS, cells were distributed into a 384-well plate with assay buffer (Tag-lite medium). To construct the inhibition dose–response curves for simufilam and Aβ_42_, varying concentrations of simufilam or unlabeled Aβ_42_ were added to corresponding wells, followed by 10 nM Aβ_42_-FAM (5-carboxyfluorescein-labeled Aβ_42_) in a final reaction volume of 14 μL. Plates were incubated 2–4 h at room temperature and read in a Tecan F500 plate reader (Tecan; Männedorf, Switzerland) with the following settings: donor excitation at 340 nm; 1st emission detection at 520 nm (acceptor) and 2nd emission at 620 nm (donor); delay: 150 μs; integration time: 500 μs. Data are expressed as the acceptor/donor ratio normalized as % of maximal Aβ_42_-FAM binding (maximal TR-FRET ratio = 100%). Specific binding is defined as the difference between total binding and non-specific binding in the presence of an excess of unlabeled Aβ_42_ (1 μM). 

### 4.3. Postmortem Human Brain Tissue

The postmortem brain study protocol conformed to the tenets of the Declaration of Helsinki as reflected in a previous approval by the City College of New York and the City University of New York Medical School’s human research committee. Each participant underwent a uniform clinical evaluation that included a medical history, complete neurological examination, cognitive testing including a mini mental state examination and other cognitive tests on episodic memory, semantic memory and language, working memory, perceptual speed, and visuospatial ability, as well as a psychiatric rating. AD subjects were diagnosed based on NINCDS-ADRDA criteria [65]. Frontal cortices from patients with clinically diagnosed sporadic AD and age-matched, neurotypical persons were obtained from the Harvard Brain Tissue Resource Center (HBTRC, Belmont, MA, USA) and the UCLA Brain Tissue Resource Center (UBTRC, Los Angeles, CA, USA). Both HBTRC and UBTRC are supported in part by the National Institutes of Health. The postmortem time intervals for collecting these brains were under 13 h (mean postmortem intervals for AD and control brain samples were 6.0 ± 0.9 h and 5.8 ± 0.8 h, respectively). Diagnostic neuropathological examination was also conducted on fixed sections stained with hematoxylin and eosin and with modified Bielschowsky silver staining [66] to establish any disease diagnosis according to defined criteria [67]. The presence of both neuritic (amyloid) plaques and neurofibrillary tangles in all AD brains was confirmed by Nissl and Bielschowsky staining and characterized by anti-Aβ_42_ and -neurofibrillary tangle (NFT) immunohistochemistry staining in the frontal and entorhinal cortex, as well as the hippocampus, as described [21]. Control tissues exhibited only minimal, localized microscopic neuropathology of AD (0–3 neuritic plaques/10% field and 0–6 NFTs/10% field in hippocampus). One-gram blocks from Brodmann areas 10 and/or 46 of frontal cortices were dissected from fresh frozen coronal brain sections maintained at −80 °C. Following the removal of white matter, gray matter was divided into ~50 mg blocks on dry ice and returned to −80 °C until use. 

### 4.4. In Vivo Oral Administration of Simufilam

As described [10], 4- and 8-month-old male and female wildtype E129 mice (30–35 g) from Taconic and 3xTg AD mice (containing 3 mutations: APP Swedish, MAPT P301L, and PSEN1 M146V) of stock supplied by Dr. Frank LaFerla [68] were maintained on a 12 h light/dark cycle with free access to food and water. We first determined the average daily intake of water sweetened with 0.25 g sucralose/100 mL to be ~5 mL. Mice then received either sweetened water alone or with simufilam at 22 mg/kg/d for 2 months. After decapitation, brain regions from one half of the brain were immediately frozen in liquid nitrogen and stored at −80 °C until use. Two equal samples (~5 mg) were separately processed to obtain synaptosomes (P2 fraction) as described [24] for assessments of FLNA linkage to CCR5/CD4/CXCR4 and CCL3-induced Gq/11 recruitment to CCR5. Synaptosomes were washed twice and suspended in 2 mL ice-cold oxygenated Krebs–Ringer solution (K-R: 25 mM HEPES, pH 7.4; 118 mM NaCl, 4.8 mM KCl, 25 mM NaHCO_3_, 1.3 mM CaCl_2_, 1.2 mM MgSO_4_, 1.2 mM KH_2_PO_4_, 10 mM glucose, 100 mM ascorbic acid) with protease and protein phosphatase inhibitors (Roche Diagnostics, Mannheim, Germany) and aerated for 10 min with 95% O_2_/5% CO_2_. Protein concentration was determined by the Bradford method (Bio-Rad, Hercules, CA, USA). 

### 4.5. Assessment of Cytokine Levels in Primary Human Astrocytes

Primary astrocyte cultures were prepared according to the provider (Lonza Biosciences, Basel, Switzerland). Adherent astrocytes were trypsinized by 0.25% trypsin-EDTA, collected and sub-cultured in 12-well plates (1.2 mL/well). When 80–85% confluent, cells were incubated with 100 fM, 10 pM or 1 nM simufilam or culture medium only under 5% CO_2_ for 2 h, prior to adding 1 μg/mL LPS, 10 μg/mL LTA-SA or 1 μg/mL PGN-SA for an additional 24 h. Levels of TNF-α, IL-6 and IL-1β in 200 μL culture medium were determined, with the medium as the blank. Each well was sampled twice. Biotinylated mouse monoclonal anti-TNFα, -IL-6, and -IL-1β (0.5 mg/well) were coated onto streptavidin-coated plates (Reacti-Bind NeutrAvidin high-binding capacity coated 96-well plate). Plates were washed 3 times with 200 μL ice-cold 50 mM Tris HCl (pH 7.4) and incubated at 30 °C with 100 μL culture medium for 1 h. Plates were washed 3 more times with ice-cold Tris HCl and incubated at 30 °C with 0.5 mg/well unconjugated rabbit anti-TNFα, -IL-6, and -IL-1β for 1 h. After 2 washes with ice-cold Tris HCl, each well was incubated in 0.5 mg/well fluorescein isothiocyanate (FITC)-conjugated anti-rabbit immunoglobulin G (human and mouse absorbed) for 1 h at 30 °C. Plates were again washed 3 times with ice-cold Tris HCl, and residual FITC signals were determined by a multimode plate reader (DTX880, Beckman Coulter, Irving, TX, USA). 

### 4.6. Assessment of FLNA–TLR2 Interaction in Postmortem Human Brain Tissue

Using an established method [9], levels of FLNA linkage to TLR2 were determined by co-immunoprecipitation of synaptosomes prepared from frontal cortical slices from 3 non-demented control subjects [41]. Frontal cortical slices were incubated with K-R, 100 nM Aβ_42_, 10 μg/mL LTA-SA or 1 μg/mL PGN-SA with or without 1 or 10 nM simufilam at 37 °C for 30 min. The incubation mixture (volume 0.5 mL) was aerated for 1 min every 15 min with 95% O_2_/5% CO_2_. Reactions were terminated by adding 1.5 mL ice-cold Ca^2+^-free K-R containing protease and protein phosphatase inhibitors, and slices were collected by brief centrifugation and processed to obtain synaptosomes (P2 fraction) as described previously [24]. 

Synaptosomes (200 μg) were pelleted by centrifugation, solubilized by brief sonication in 250 μL immunoprecipitation buffer (25 mM HEPES, pH 7.5; 200 mM NaCl, 1 mM EDTA, with protease and protein phosphatase inhibitors) and incubated at 4 °C with end-to-end shaking for 1 h. Following dilution with 750 μL ice-cold immunoprecipitation buffer and centrifugation (4 °C) to remove insoluble debris, the FLNA–TLR2 complexes in the lysate were isolated by immunoprecipitation with 16 h incubation at 4 °C with anti-FLNA (SC-7565; 1 μg) immobilized on protein A/G-conjugated agarose beads. Resultant immunocomplexes were pelleted by centrifugation at 4 °C. After 3 washes with 1 mL ice-cold PBS (pH 7.2) and centrifugation, the isolated FLNA–TLR2 complexes were solubilized by boiling for 5 min in 100 μL SDS-polyacrylamide gel electrophoresis (PAGE) sample preparation buffer (62.5 mM Tris-HCl, pH 6.8; 10% glycerol, 2% SDS; 5% 2-mercaptoethanol, 0.1% bromophenol blue). The TLR2 contents in 50% of the anti-FLNA immunoprecipitates were determined by immunoblotting with mouse monoclonal anti-TLR2 (SC-166900). Blots were then stripped and re-probed with monoclonal anti-FLNA (SC-271440) to ascertain equal immunoprecipitation and loading. 

### 4.7. Assessment of FLNA–CCR5/CD4/CXCR4 Interaction in Postmortem Human Brain and Transgenic AD Mouse Brain

Using the same method [9], the linkage of FLNA with CCR5, CXCR4 and CD4 in synaptosomes from Aβ_42_-incubated frontal slices from 11 sets of age- (66–92 years) and postmortem interval (2–13 h)-matched control and AD subjects (4 females/7 males) with and without 1 nM simufilam were immunoprecipitated with immobilized anti-FLNA (SC-7565). In the experiments using postmortem human brains, frontal cortical slices were incubated with K-R or 1 nM simufilam at 37 °C for 30 min. The incubation mixture (volume 0.5 mL) was aerated for 1 min every 15 min with 95% O_2_/5% CO_2_. Reactions were terminated by adding 1.5 mL ice-cold Ca^2+^-free K-R containing protease and protein phosphatase inhibitors, and slices were collected by brief centrifugation and processed to obtain synaptosomes (P2 fraction) as described [24]. 

Synaptosomes (200 μg) prepared from K-R- or simufilam-incubated postmortem cortical slices or from wildtype or transgenic mice were pelleted by centrifugation, solubilized by brief sonication in 250 μL immunoprecipitation buffer (described above) and incubated at 4 °C with end-to-end shaking for 1 h. Following dilution with 750 μL ice-cold immunoprecipitation buffer and centrifugation (4 °C) to remove insoluble debris, the FLNA–CCR5/CD4/CXCR4 complexes in the lysate were isolated by immunoprecipitation with 16 h incubation at 4 °C with anti-FLNA (1 μg) immobilized on protein A/G-conjugated agarose beads (anti-FLNA for postmortem human brain: SC-7565; for mice: SC-17749). The immunocomplexes were pelleted by centrifugation at 4 °C. After 3 washes with 1 mL ice-cold PBS (pH 7.2) and centrifugation, the isolated FLNA–CCR5/CD4/CXCR4 complexes were solubilized by boiling for 5 min in 100 mL SDS-PAGE sample preparation buffer. Levels of CCR5, CD4, and CXCR4 IRβ in 50% of the anti-FLNA immunoprecipitates were determined by immunoblotting with mouse monoclonal CCR5 (SC-17833), CD4 (SC-19641), and CXCR4 (SC-53534) antibodies, sequentially. A separate set of blots was probed with monoclonal anti-FLNA (SC-271440) to validate equal immunoprecipitation efficiency and loading. 

### 4.8. CCL3-Stimulated Gq/11 Recruitment to CCR5 in Synaptic Membranes

Synaptosomes (P2 fraction) were prepared from snap-frozen parietal cortices of vehicle- and simufilam-treated wildtype and transgenic mice as previously described [69,70]. To further purify synaptosomal factions, the synaptosome-rich P2 fraction was washed twice in 1 mL oxygenated ice-cold K-R with protease and protein phosphatase inhibitors. To obtain membranous fractions of the synaptosomes, washed synaptosomes were sonicated for 10 sec on ice in 0.5 mL hypotonic homogenization solution (25 mM HEPES, pH 7.4; 12 mM NaCl, 0.5 mM KCl, 2.5 mM NaHCO_3_, 0.1 mM CaCl_2_, 0.1 mM MgSO_4_, 0.1 mM KH_2_PO_4_, 1 mM glucose, 10 mM ascorbic acid, protease and protein phosphatase inhibitors). Samples were then centrifuged at 50,000× *g* for 30 min. The resultant synaptic membrane pellet was resuspended in 0.5 mL K-R, and protein concentrations were determined by the Bradford method. These synaptic membranes were stimulated with the CCR5 ligand CCL3, and levels of CCR5-coupled Gq/11 were determined using an established method [71]. 

Synaptic membranes (100 μg) were incubated in 200 μL K-R or in 10 nM CCL3 at 37 °C for 10 min. The reaction was stopped by adding 20 mM MgCl_2_ and centrifuging. The pelleted synaptic membranes were solubilized by brief sonication (10 sec, 50% output, Fisher Scientific, Waltham, MA, USA) on ice in 250 μL immunoprecipitation buffer and solubilized by adding 0.5% digitonin, 0.2% sodium cholate and 0.5% NP-40 and incubated at 4 °C with end-to-end shaking for 1 h. Following dilution with 750 μL ice-cold immunoprecipitation buffer and centrifugation at 4 °C to remove insoluble debris, the resultant lysate was used to measure levels of CCR5-associated Gq/11 by the quantities of Gαq/11 in the anti-CCR5 immunoprecipitates. Briefly, the CCR5-Gq/11 complexes in the lysate were isolated by immunoprecipitation with 16 h incubation at 4 °C with 1 μg anti-CCR5 (SC-17833) immobilized on protein A/G-conjugated agarose beads. The immunocomplexes were pelleted by centrifugation at 4 °C. After 3 washes with 1 mL ice-cold PBS (pH 7.2) and centrifugation, the isolated CCR5-Gq/11 complexes were solubilized by boiling for 5 min in 100 μL SDS-PAGE sample preparation buffer. Levels of Gαq/11 in 50% of the anti-CCR5 immunoprecipitates were determined by immunoblotting with mouse anti-Gαq/11 (SC-515689). The other 50% of the anti-CCR5 immunoprecipitates were run on separate blots probed with monoclonal anti-CCR5 (SC-17833) to validate equal immunoprecipitation efficiency and loading. 

### 4.9. Statistics

For the TR-FRET assay, nonlinear fitting of the concentration curve and calculation of pIC_50_ was performed using GraphPad Prism software version 9. FLNA–receptor linkages in postmortem brain tissue were analyzed by two-way ANOVA with diagnosis (AD/control) and treatment (simufilam/vehicle) as factors with post hoc *t*-tests for pair-wise comparisons. Student’s *t*-test was used for all other statistical analyses. 

## 5. Conclusions

FLNA, in an altered conformation, is a deviant receptor-associated protein critical to AD pathology. Simufilam’s disruption of the aberrant FLNA linkage to α7nAChR reduces Aβ_42_’s binding to and pathogenic signaling via this receptor, thereby restoring healthy α7nAChR neurotransmission. Simufilam’s disruption of deviant FLNA linkages to multiple inflammatory receptors suppresses neuroinflammation induced by these receptors. The dissociation of FLNA from all these receptors is coincident with simufilam’s reversal of an altered conformation of FLNA, as indicated by isoelectric focusing points. It is not surprising that an altered conformation, inducible by soluble Aβ_42_, would lead to aberrant protein interactions. Alternatively, Aβ_42_-induced aberrant protein interactions could induce the altered conformation. 

By binding a single protein target, simufilam reduces a predominant neurodegeneration pathway and multiple neuroinflammatory signaling pathways of soluble amyloid and potentially other inflammatory ligands in AD. A multi-pronged therapeutic approach, whether by agents with multiple mechanisms or by drug combinations, may be necessary to treat this devastating disease. 

## Data Availability

The data presented in this work are presented in the article.
